# A First-Generation Multi-Functional Cytokine for Simultaneous Optical Tracking and Tumor Therapy

**DOI:** 10.1371/journal.pone.0040234

**Published:** 2012-07-11

**Authors:** Shawn Hingtgen, Randa Kasmieh, Elizabeth Elbayly, Irina Nesterenko, Jose-Luiz Figueiredo, Rupesh Dash, Devanand Sarkar, David Hall, Dima Kozakov, Sandor Vajda, Paul B. Fisher, Khalid Shah

**Affiliations:** 1 Molecular Neurotherapy and Imaging Laboratory, Massachusetts General Hospital, Harvard Medical School, Boston, Massachusetts, United States of America; 2 Department of Radiology, Massachusetts General Hospital, Harvard Medical School, Boston, Massachusetts, United States of America; 3 Department of Neurology, Massachusetts General Hospital, Harvard Medical School, Boston, Massachusetts, United States of America; 4 Harvard Stem Cell Institute, Harvard University, Cambridge, Massachusetts, United States of America; 5 Department of Human and Molecular Genetics, VCU Institute of Molecular Medicine, VCU Massey Cancer Center, Virginia Commonwealth University, School of Medicine, Richmond, Virginia, United States of America; 6 Department of Biomedical Engineering, Boston University, Boston, Massachusetts, United States of America; City of Hope National Medical Center and Beckman Research Institute, United States of America

## Abstract

Creating new molecules that simultaneously enhance tumor cell killing and permit diagnostic tracking is vital to overcoming the limitations rendering current therapeutic regimens for terminal cancers ineffective. Accordingly, we investigated the efficacy of an innovative new multi-functional targeted anti-cancer molecule, SM7L, using models of the lethal brain tumor Glioblastoma multiforme (GBM). Designed using predictive computer modeling, SM7L incorporates the therapeutic activity of the promising anti-tumor cytokine MDA-7/IL-24, an enhanced secretory domain, and diagnostic domain for non-invasive tracking. *In vitro* assays revealed the diagnostic domain of SM7L produced robust photon emission, while the therapeutic domain showed marked anti-tumor efficacy and significant modulation of p38MAPK and ERK pathways. *In vivo*, the unique multi-functional nature of SM7L allowed simultaneous real-time monitoring of both SM7L delivery and anti-tumor efficacy. Utilizing engineered stem cells as novel delivery vehicles for SM7L therapy (SC-SM7L), we demonstrate that SC-SM7L significantly improved pharmacokinetics and attenuated progression of established peripheral and intracranial human GBM xenografts. Furthermore, SC-SM7L anti-tumor efficacy was augmented *in vitro* and *in vivo* by concurrent activation of caspase-mediated apoptosis induced by adjuvant SC-mediated S-TRAIL delivery. Collectively, these studies define a promising new approach to treating highly aggressive cancers, including GBM, using the optimized therapeutic molecule SM7L.

## Introduction

Successful management of many terminal cancer types has not been achieved primarily because many conventional anti-cancer therapies lack tumor specificity and exhibit poor or inadequate pharmacokinetics that result in high toxicity to normal tissue, limited bioavailability, and subsequently ineffective tumor cell killing. Recently, the development of multifunctional therapeutics offers unprecedented potential to overcome the limitations of current anti-cancer therapies [1]–. These powerful anti-cancer therapies combine targeting specificity, optimized pharmacokinetics, and diagnostic imaging capacity into a single agent. When used in combination with effective delivery systems, these multifunctional molecules have the potential to specifically target tumors with high levels of localized therapies that can be monitored in real-time by non-invasive imaging. Although widely applicable, multifunctional molecules are especially well suited for treatment of tumors in the brain where additional obstacles such as the blood-brain-barrier make treating aggressive malignancies particularly challenging [2]. Currently, no effective treatment has been identified for the most common primary brain tumor, Glioblastoma multiforme (GBM), and median survival rates remain at approximately 1 year [6]. Therefore, new multifunctional molecules offer a huge potential for successfully managing terminal cancer types that include GBM.

To date, the majority of multifunctional agents are synthetic nanoscale devices or nanoparticles chemically engineered with tumor targeting and/or imaging components [2], [3]. Novel multifunctional DNA-encoded protein-based molecules offer unique advantages over these devices, particularly for monitoring secreted therapeutics delivered through unique cell-based applications. Despite their promise and wide applicability, few multifunctional DNA-based molecules exist. Furthermore, the fusion of multiple domains to create multifunctional DNA-based molecules decreases the activity of the domains resulting in reduced diagnostic and/or therapeutic activity compared to the parental molecules [1], [7]. Consequently, creating molecules that retain or enhance the functionality of the multiple domains remains a significant challenge. Melanoma differentiation associated gene-7/interleukin-24 (MDA-7/IL-24) is a naturally occurring anti-cancer cytokine that induces a strong tumor suppressive effect in various cancer cell types while sparing normal cells [8], [9]. Located on chromosome 1q32 [9], the anti-tumor effects of MDA-7/IL-24 have been validated on cancer cell lines that include melanoma [10], breast cancer [8], [9], [11], [12], prostate cancer [8], [9], [11], [12], hepatocellular carcinoma [13], GBM [14]–[16] and others [8], [9], [11], [12], [17]. In preclinical studies, MDA-7/IL-24 therapy has been shown to slow tumor progression both *in vitro* and *in vivo* [15], [16]. The effects of extracellular MDA-7/IL-24 are mediated by binding to IL-20Rα/β receptors on tumors cells, inducing modulation of the ERK, JNK, MAPK pathways to induce anti-tumor effects [15]. Based on the potent anti-tumor activity and receptor-targeted specificity of MDA-7/IL-24, we reasoned that a novel and highly effective multifunctional anti-cancer molecule could be created by re-engineering of wild-type MDA-7/IL-24. However no studies to date have engineered variants of MDA-7/IL-24 that alter it’s extracellular secretion, functionality, or introduce diagnostic properties to the parental molecule. We further speculated that both the pharmacokinetics and anti-cancer efficacy of the novel multifunctional MDA-7/IL-24 variant would be improved by delivery via engineered stem cells. Our laboratory and others have shown that engineered stem cells display inherent tumor-tracking properties that allow local on-site secretion of therapies that increases delivery time and decrease non-specific tissue binding [1], [18]–[21].

In the current study, we engineered and validated a new multifunctional molecule SM7L, and tested the anti-tumor efficacy of stem cell-delivered SM7L therapy using models of the deadly brain cancer GBM both *in vitro* and *in vivo*. SM7L was designed to incorporate: 1) the anti-tumor domain of MDA-7/IL-24 to introduce cancer cell-specificity and robust therapeutic activity, 2) light-emitting luciferase domain to impart diagnostic imaging capacity, and 3) altered secretion sequence to optimize extracellular delivery. Our results demonstrate the multifunctional properties of SM7L and the efficacy of stem cell-mediated SM7L therapy against highly aggressive brain tumor GBM. These results define a promising new approach to treating highly aggressive cancers using the multifunctional anti-cancer molecule SM7L.

## Results

### Design and Validation of SM7L

**Figure 1:**
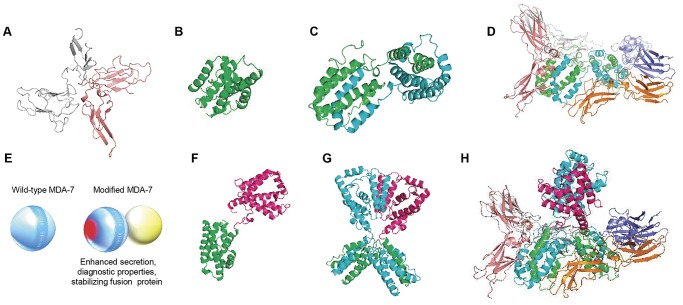
Predictive computer modeling of wild-type MDA-7/IL-24 and optimized SM7L. Predictive space-filling models of IL-20α/β receptor (A), MDA-7/IL-24 monomer (B), MDA-7/IL-24 dimer (C), and dimerized MDA-7/IL-24 binding to IL-20α/β receptor (D). (E) Representative models of wild-type MDA-7/IL-24 and the modifications to optimize the molecule. These include modification of the secretion sequence (in red) and C-terminal diagnostic fusion (yellow). (F-H) Space-filling models demonstrating the predicted structure of the modified MDA-7/IL-24 fusion protein SM7L. (F) Representative computer model of SM7L monomer containing MDA-7/IL-24 (green) and C-terminal luciferase fusion (pink). (G) Computer model showing a SM7L dimer. (H) Space filling model of SM7L dimmer interacting with IL-20α/β receptor.

Predictive molecular modeling was used to interrogate the structure of wild type-MDA-7/IL-24, and provide a rational basis for designing a potentially optimized molecule. The “bystander” antitumor effects of MDA-7/IL-24 protein are mediated by binding to the IL-20R1/IL-20R2 and IL-22R1/IL-20R2 (IL-20Rα/β) receptors (Fig. 1A) on the surface of cells [22]–[26]. Wild-type-MDA-7/IL-24 monomers (Fig. 1B) aggregate to form dimers (Fig. 1C), which then bind and activate defined molecular pathways downstream of IL-20Rα/β receptors (Fig. 1D) to inhibit tumor cell proliferation and survival. We hypothesized that an ideal MDA-7/IL-24-based molecule for cell delivery would contain: 1) maximal extracellular secretion for optimal delivery of this therapeutic; 2) diagnostic properties for rapid non-invasive tracking; and 3) a c-terminal fusion which would increase stability of the molecule and improve functionality (Fig. 1E). Based on these predictions, we designed SM7L (Fig. 1F) that contained a modified secretion sequence derived from Flt-3 ligand and C-terminal fusion of Gaussia luciferase to increase both stability and introduce diagnostic properties. Molecular modeling suggested that this molecule would retain the ability to efficiently dimerize (Fig. 1G) and interact with cell-surface IL-20Rα/β receptors (Fig. 1H). Furthermore, the modeling suggested the C-terminal fusion could increase stability of the molecule in the binding pocket leading to enhanced receptor activation and greater anti-tumor effects.

### Characterizing Stem Cell-Delivered SM7L *In Vitro*

**Figure 2:**
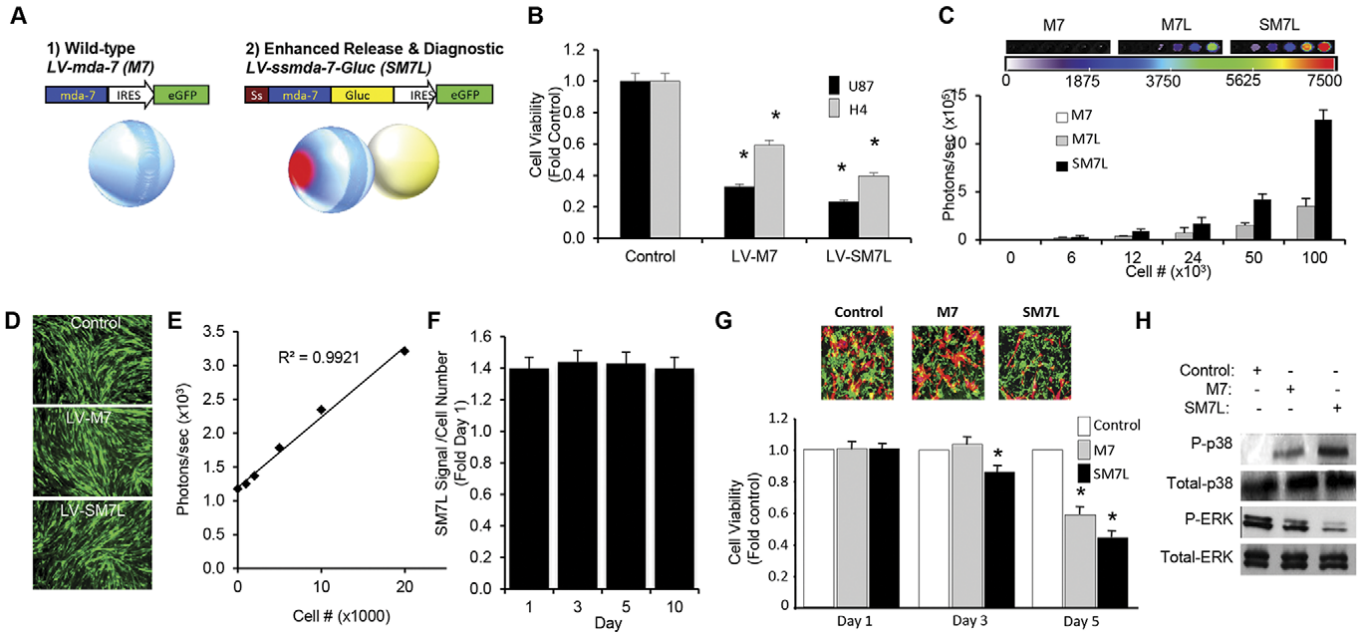
*In vitro* characterization of stem cell-delivered SM7L. (A) Lentiviral transfer vectors were engineered to encode: (1) wild-type MDA-7/IL-24 (M7) and (2) SM7L. The modified secretion sequence is shown in red and the luciferase fusion is shown in yellow. All MDA-7/IL-24 variants were cloned upstream of IRES-GFP. (B) Summary data demonstrating the effects of SM7L on U87 or H4 human GBM cells. Tumor cells were transduced with lentiviral vectors encoding SM7L, M7, or GFP and the therapeutic activity of each variant was determined 5 days post-transduction. (C) Representative BLI images and summary data showing the extracellular levels of SM7L, wild-type M7, or a wild-type M7 diagnostic fusion lacking a modified secretion sequence (M7L). 293 T cells were transduced with equal MOI of LV-SM7L, LV-M7, or LV-M7L and seeded at increasing cell numbers in 96-well plates. Forty-eight h later, equal volumes of media were transferred to new wells and BLI imaging was performed on media. (D) Representative images of stem cell cells transduced with LV encoding GFP (control), M7, or SM7L. (E) Summary graph demonstrating the linear correlation between mNSC-SM7L cell number and photon emission. mNSC-SM7L were seeded at increasing cell numbers (0.1–2×10^4^ cells), combined with coelenterazine 15 µg, and photon emission was determined in a luminometer. (F) Summary data demonstrating the stability of SM7L expression by mNSC. mNSC were transduced with LV-SM7L, and 1, 3, 5, and 10 days post-transduction, cells (1×10^5^ cells) were collected, combined with coelenterazine (15 µl), and SM7L photon emission was determined using a luminometer. Data is expressed relative to cell number determined by luciferase-based assay. (G) Representative images and summary graphs of cell viability assays performed on U87 human GBM co-cultured with mNSC-GFP, mNSC-M7, or mNSC-SM7L. U87-Fluc-mcherry GBM cells were seeded in 96 well plates. 24 h later, control or mNSC secreting wild-type or SM7L were overlayed on the cells. Three and five days later, Fluc imaging was performed to determine the effects of stem cell-delivered M7 or SM7L on GBM cell growth. (H) Western blot performed on lysates from U87 GBM cells treated with conditioned media from control, mNSC-M7 or mNSC-SM7L. Cell lysates from treated cells were immunoblotted using antibodies against phosphorylated and non-phosphorylated p38 MAPK or ERK. In all panels, *p<0.05 vs. control, and experiments were performed in triplicate with mean and SD reported.

To allow easy and efficient transduction of cells with SM7L, we next engineered lentiviral vectors encoding wild-type MDA-7/IL-24 (M7) and the optimized SM7L (Fig. 2A). To assess the anti-tumor activity of LV-SM7L, we transduced U87MG (high IL-20Rα/β, Fig. S1) or H4 (low IL-20Rα/β) human GBM cells with LV-SM7L, LV-M7 or control. As shown in figure 2B, cell viability assays performed 5 days after transduction revealed LV-SM7L significantly reduced cell viability in both cell lines, and to comparable or greater levels than wild-type LV-M7. Due to the robust response of U87 to SM7L treatment, this cell line was used for all further experiments assessing therapeutic efficacy in this study. Revealing the diagnostic properties of SM7L, bioluminescence imaging (BLI) of media from cells transduced with LV-SM7L showed robust light emission that was absent in media from control wild-type LV-M7 transduced cells (Fig. 2C), and showed significantly higher extracellular photon emission than cells expressing a MDA-7/IL-24-Gaussia luciferase fusion lacking the Flt-3 signal sequence suggesting increased extracellular secretion due to the modified secretion sequence in SM7L (Fig. 2C).

Next, we assessed the potential of utilizing the optimized SM7L to create a novel multifunctional cell-based therapeutic for simultaneous non-invasive tracking and tumor killing. Primary mouse neural stem cells (mNSC) were engineered with LV encoding SM7L (mNSC-SM7L), wild-type MDA-7/IL-24 (mNSC-M7), or control (mNSC-GFP), and transduction was confirmed by fluorescence imaging (Fig. 2D). *In vitro* BLI showed a direct correlation between mNSC-SM7L cell number and photon emission (Fig. 2E), while longitudinal BLI revealed a stable secretion of SM7L by stem cells through 10 days of culture (Fig. 2F). To determine the anti-GBM effects of stem cell-delivered SM7L and underlying molecular pathways, we performed co-culture analysis of mNSC-M7, mNSC-SM7L, or mNSC-GFP with U87 cells that were engineered to express the fusion of mCherry and Firefly luciferase (FLuc). Quantitative FLuc imaging on day 3 and day 5 of co-culture revealed mNSC-SM7L significantly inhibited U87 proliferation (Fig. 2G), and these results were verified by similar findings in U251 and primary patient-derived GBM8 tumor lines (Fig. S2). Western blot analysis showed robust activation of p38MAPK and marked down regulation of ERK signaling by mNSC-SM7L treatment in U87 (Fig. 2H). Taken together these results demonstrate that the optimized variant, SM7L, expands the functionality of wild-type MDA-7/IL-24 by incorporating diagnostic tracking properties, promoting greater extracellular secretion, and increasing anti-glioma activity.

### Stem Cell-based Delivery Improves the Pharmacokinetics of SM7L Delivery and Attenuates GBM Progression *In Vivo*

**Figure 3:**
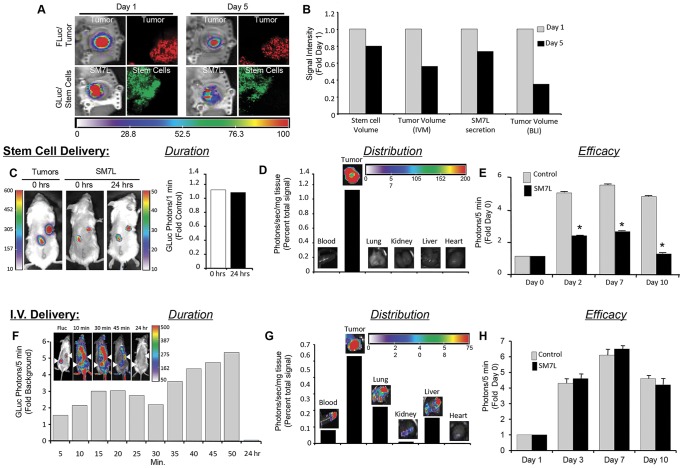
mNSC-SM7L improves the pharmacokinetics of delivery and enhances anti-GBM efficacy *in vivo* . (A–B) Representative images and summary graph showing *in vivo* imaging of multiple events in stem cell delivery of SM7L. mNSC-SM7L were implanted around established U87Fluc-mCherry tumors in subcutaneous skin flap window chambers. Intravital microscopy was used to visualize stem cell (green) and tumor (red) volumes at cellular resolution 1 and 5 days post-implantation (A). Simultaneously, Fluc and GLuc imaging was used to monitor changes in tumor volume (Fluc) and SM7L (GLuc) secretion respectively (A). Fluorescent intensities and BLI photon emission was then quantified to reveal stem cell volume, tumor volumes, and SM7L levels (B). In the panels, tumors = Fluc, red; stem cells = green; SM7L secretion = GLuc. (C–H) Summary of experiments to visualize differences in pharmacokinetics of stem cell-delivered and intravenous (IV) injection of SM7L. (C) Representative Fluc (tumor) and GLuc (SM7L) images and summary data showing SM7L levels delivered by engineered stem cells implanted around established U87-Fluc tumors at 0 and 24 h post-implantation (n = 3). (D) Ex vivo analysis of stem cell-delivered SM7L biodistribution performed by GLuc bioluminescent imaging of multiple excised tissues. (E) Summary graph of Fluc imaging performed on days 0, 2, 7, and 10 showing mNSC-SM7L inhibition of tumor progression compared to tumors treated with control stem cells. (F–H) *In vivo* imaging of conditioned medium containing SM7L injected by IV infusion. Following implantation of FLuc-positive tumors (F), a bolus of media containing SM7L was injected IV followed immediately by coelenterazine. Images were captured at 5–50 mins and 24 h post-injection (F, n = 3). Representative Fluc (tumor) and GLuc (SM7L) images and summary graphs revealing levels (F) and distribution in excised tissue (G) are shown. (H) Fluc imaging was performed on days 1, 3, 7 and 10 post-injection to determine tumor volumes.

We next determined which events in stem cell-mediated delivery to tumors could be tracked by a combination of multimodality imaging and diagnostic markers utilizing the additional diagnostic properties of SM7L. To this end, GFP-labeled stem cells secreting SM7L were implanted adjacent to established U87 glioma cells expressing mCherry-FLuc in skin fold window chambers (Fig. 3A). High-resolution intravital microscopy performed on day 1 revealed the distribution of green SM7L-secreting stem cells adjacent to red U87 gliomas, and these distinct populations could be followed through day 5 (Fig. 3A). Monitoring of SM7L levels by BLI revealed robust secretion at day 1 that had declined slightly by day 5 (Fig. 3A–B, GLuc), while FLuc imaging showed GBM volumes could be simultaneously monitored to permit assessment of therapeutic efficacy (Fig. 3A–B, FLuc). Together, these results show the combination of longitudinal molecular imaging and diagnostic properties of SM7L permits non-invasive simultaneous monitoring of SM7L levels, tumor volumes, stem cell volumes, and cell distribution.

We utilized a molecular imaging approach to next investigate the *in vivo* pharmacokinetics and therapeutic efficacy of SM7L delivered by engineered stem cells. In the clinic setting, therapeutic stem cells are typically injected directly into tumors. When mNSC-SM7L were implanted around established U87-FLuc tumors to mimic clinical cell engrafts typical of stem cell-based treatments, bioluminescence imaging revealed a concentrated and stable SM7L signal that co-localized with the established tumor and showed no change in levels through 24 h (Fig. 3C). *Ex vivo* tissue analysis showed SM7L was present in the excised tumor, but absent from other organs (Fig. 3D). Importantly, the constant and localized delivery of SM7L by stem cells lead to a marked reduction in tumor volumes as early as day 2, assessed by Fluc imaging (Fig. 3E). We next compared the pharmacokinetics of stem cell-mediated SM7L delivery to purified SM7L delivered by intravenous (IV) infusion to mimic the systemic or IV delivery typically utilized for most clinical chemotherapies and therapeutic proteins. Serial BLI showed the IV bolus of concentrated media containing SM7L lead to widely-distributed levels shortly after injection that were rapidly cleared by 24 h (Fig. 3F). *Ex vivo* tissue analysis showed SM7L present in the blood, lung, kidney, liver, as well as the tumor (Fig. 3G). FLuc imaging of tumor volumes showed that intravenous SM7L had no significant effect on tumor volumes (Fig. 3H). These results document that mNSC-SM7L improves delivery and efficacy of MDA-7/IL-24 therapy, and the diagnostic properties of SM7L provide previously unavailable insights into real-time pharmacokinetics of this cytokine.

**Figure 4:**
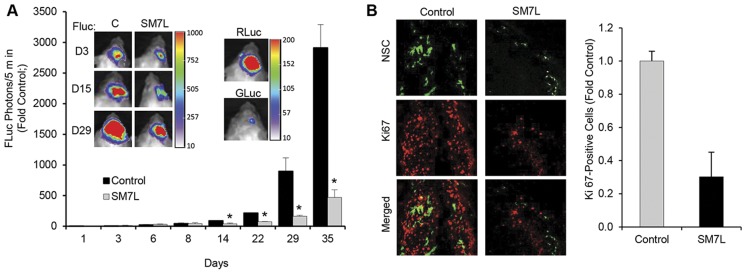
mNSC-SM7L prolongs SM7L delivery and attenuates GBM progression. (A) Representative FLuc bioluminescent images and summary data of mice implanted intracranially with mNSC transduced with LV-GFP-RLuc (n = 6) or SM7L (n = 6) and U87-Fluc GBM cells. FLuc bioluminescence imaging was performed from day 1 to day 35 to monitor tumor progression. On day 29, Rluc imaging was performed to determine stem cell volume and GLuc imaging was performed to confirm SM7L secretion. (B) Immunohistochemistry with antibodies against Ki67 and summary data performed on sections from brains treated with control and mNSC-SM7L 29 days post-implantation. Representative images show brain sections containing mNSC (green) or Ki67 staining (red). In all panels, *p<0.05 vs. control, and mean and SD are reported.

We next tested the efficacy of mNSC-SM7L in an intracranial mouse model of GBM. U87-FLuc GBM cells were implanted with mNSC-GFP-RLuc or mNSC-SM7L in the frontal lobe of mice. Dual Fluc/Gluc and Fluc/Rluc imaging was used to track tumor volumes/SM7L expression and tumor volumes/stem cell fate respectively. As shown in figure 4A, longitudinal tracking of tumor volumes by Fluc imaging revealed significant inhibition of GBM growth by mNSC-SM7L compared to control-treated GBMs by day 14, and this reduction in tumor progression persisted through 35 days. RLuc and Gluc imaging performed on day 29 confirmed the presence of both stem cells and SM7L 4 weeks post-treatment (Fig. 4A). Post-mortem immunohistochemical staining confirmed the difference in tumor volumes and revealed a significant reduction in Ki-67-positive GBM cells (Fig. 4B). Together, these results provide strong evidence of the ability of mNSC-SM7L to effectively inhibit growth of GBM *in vivo*.

### SM7L and Concomitant Cytotoxic Treatment Enhance GBM Cell Killing *In Vitro* and *In Vivo*

**Figure 5:**
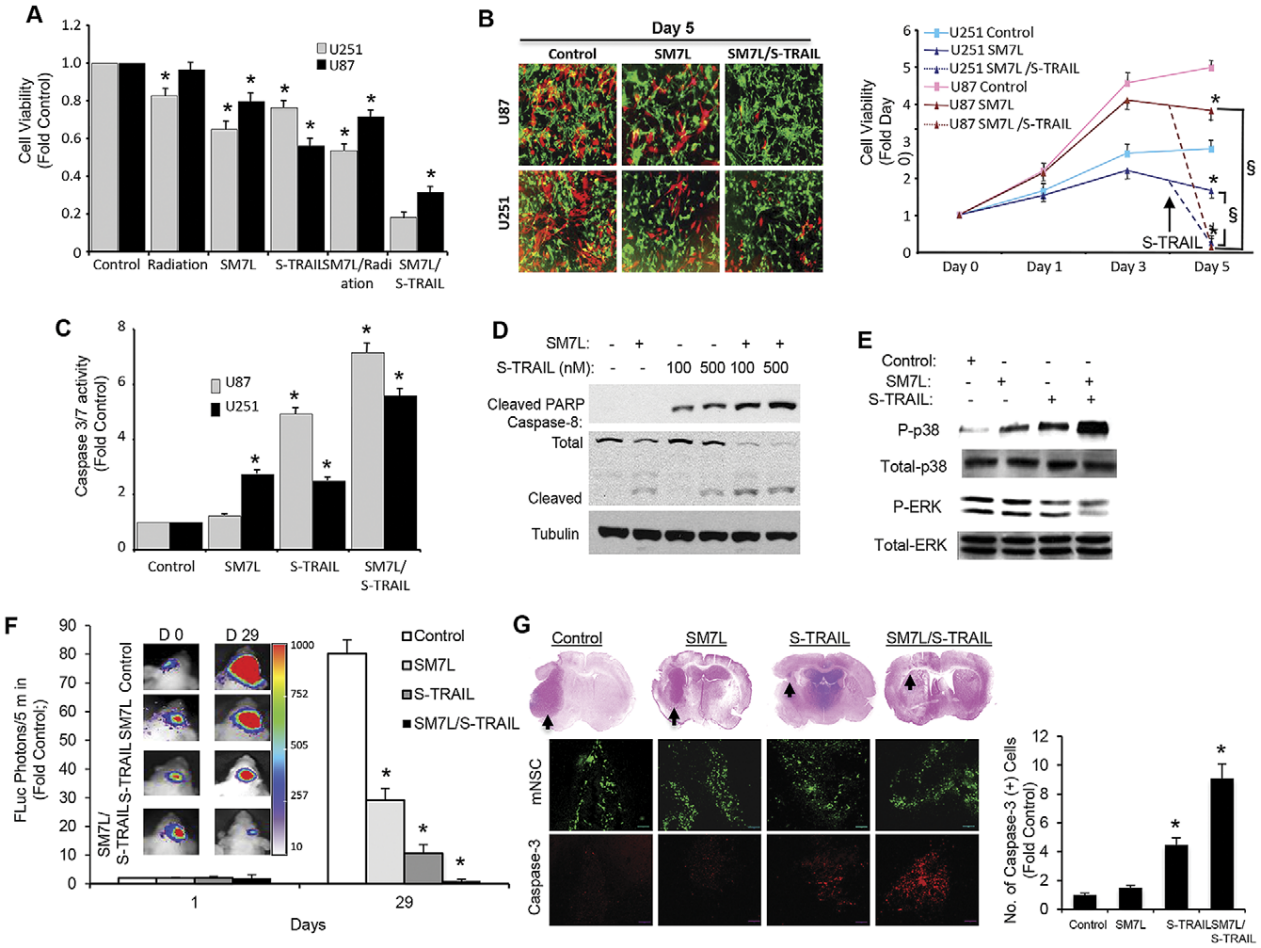
Combined targeting of cytostatic and cytotoxic pathways with stem cell delivered SM7L and S-TRAIL improves anti-GBM efficacy *in vitro* and *in vivo*. (A) Summary graph of the viability of U251 and U87 GBM cells pre-treated with radiation (4 Gy) and SM7L or SM7L followed by concentrated S-TRAIL. U251 and U87 GBM cells were incubated with SM7L or irradiated with 4 Gy., and treated or untreated cells were incubated with S-TRAIL (200 ng) overnight, and cell viability was determined by luciferase-based assay. (B) Summary data and representative images demonstrating the effects on cell viability of U251 and U87 GBM cells co-cultured with stem cell secreting SM7L or SM7L/S-TRAIL. Control or SM7L mNSC were overlayed on Fluc-positive GBM cells. Four days later, mNSC-STRAIL were seeded onto a subset of mNSC-SM7L and cell viability was determined by luciferase assay 24 h later. (C) Caspase 3/7 activation assay performed on U87 and U251 cells treated with conditioned media containing SM7L, S-TRAIL, SM7L/S-TRAIL, or control. (D-E) Western blot and summary graph performed on U87 and U251 cells treated with conditioned media containing SM7L and/or S-TRAIL. Cell lysates were collected and immunoblotted with antibodies against cleaved PARP, total and cleaved caspase-8 (D) as well as total and phosphorylated p38MAPK or ERK (E). (F) Representative images and summary data demonstrating the effects of stem cell-delivered SM7L or S-TRAIL monotherapy or SM7L/S-TRAIL concomitant therapy delivered by dual secreting stem cells on the progression intracranial U87-Fluc human GBMs. mNSC-GFP (control, n = 10), mNSC-SM7L (n = 10), mNSC-S-TRAIL (n = 8), or double secreting mNSC-SM7L/S-TRAIL (n = 8) were implanted together with U87-Fluc GBM cells in the frontal lobe of mice. Serial Fluc bioluminescence imaging was performed on days 0 and 29 to monitor therapeutic efficacy. (G) Representative photomicrographs and summary graph of coronal brain sections stained with H&E and corresponding fluorescence immunostaining showing mNSC distribution (GFP) or the levels caspase-3 (red, summary graph) in mice treated with mNSC-GFP (control), mNSC-SM7L, mNSC-S-TRAIL, or double secreting mNSC-SM7L/S-TRAIL. In all panels, *p<0.05 vs. control; §p<0.05 vs. SM7L, and experiments were performed in triplicate with mean and SD reported.

**Figure 6:**
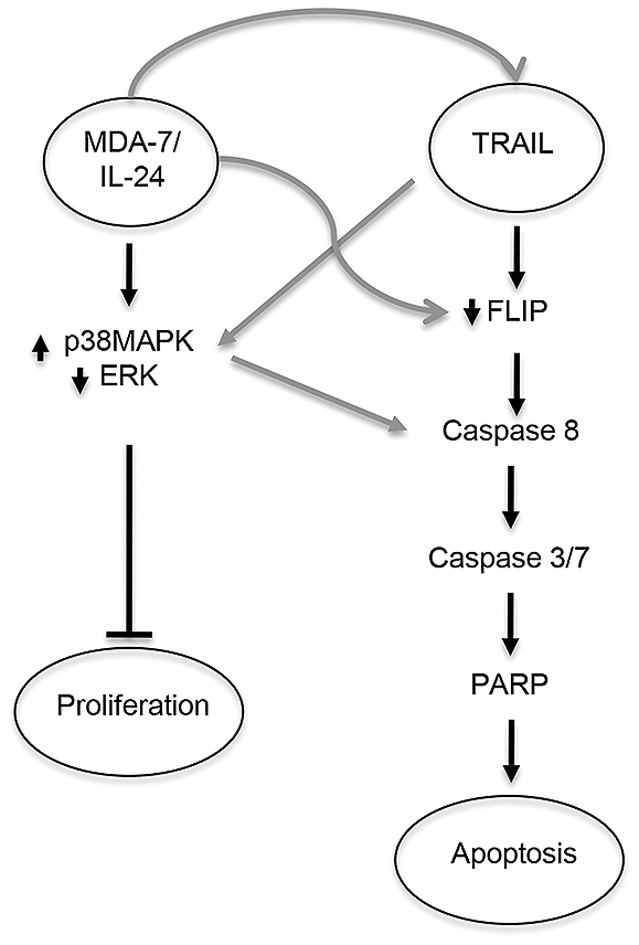
Proposed signaling changes following combined SM7L/S-TRAIL therapy. Schematic simplified overview of pathways proposed to mediate the anti-tumor effects of MDA-7/IL-24 and TRAIL combination therapy. MDA-7/IL-24 primarily induces cytostatic effects through alterations in p38MAPK and ERK signaling. TRAIL induces apoptosis via activation of the caspase cascade. Elevation in these pathways were observed following combination treatment. Speculative points of cross-talk are labeled in grey. MDA-7/IL-24 up-regulates the levels of TRAIL in certain cell lines and down-regulates the TRAIL inhibitor FLIP. TRAIL has been reported to downregulate ERK in GBM cell lines leading to increased caspase activation.

Despite the ability of mNSC-SM7L to inhibit GBM progression, residual GBM volumes were still detected at the termination of mNSC-SM7L treatment. To strengthen SM7L-based therapy we next investigated the efficacy of augmenting the cytostatic effects of SM7L with agents that induce caspase-mediated cell death that included radiation or a secreted variant of the potent pro-apoptotic therapeutic protein TNF-α-related apoptosis-inducing ligand (S-TRAIL). To investigate GBM therapy via activation of multiple anti-tumor pathways, U87 (SM7L-sensitive, TRAIL sensitive) and U251 (SM7L-sensitive; TRAIL semi-resistant [27]) GBM cells were treated with: 1) SM7L; 2) radiation (4 Gy); 3) S-TRAIL (200 ng); 4) radiation and SM7L; or 5) SM7L and S-TRAIL (200 ng). As shown in the summary data (Fig. 5A), the combination of SM7L and S-TRAIL lead to the greatest reduction in viability of both GBM cell lines (U251–82%; U87–71%; Fig. 5A). Additionally, while mNSC-SM7L co-cultured with U87 or U251 significantly slowed tumor cell proliferation, the addition of mNSC secreting S-TRAIL (mNSC-S-TRAIL) converted the cytostatic effects into a rapid reduction in GBM viability (Fig. 5B). Providing insight into the molecular mechanism, SM7L/S-TRAIL co-treatment lead to robust caspase 3/7 activation (Fig. 5C) and cleavage of caspase-8 and PARP (Fig. 5D), effects that were not observed by SM7L treatment alone (Fig. 5C–D). Interestingly, the addition of S-TRAIL to SM7L-treated GBM increased p38MAPK activation and ERK suppression to a greater extent than SM7L alone (Fig. 5E). Taken together, these results reveal that the combination of SM7L and S-TRAIL simultaneously target both cell proliferation and apoptosis pathways to increase GBM cell killing (Fig. 6).

To assess the *in vivo* efficacy of combined SM7L and S-TRAIL therapy delivered by stem cells, we engineered double secreting mNSC (mNSC-SM7L/S-TRAIL). U87-FLuc human GBM cells were implanted intracranially in mice with control mNSC-GFP, mNSC-SM7L, mNSC-S-TRAIL, or the double secreting mNSC-SM7L/S-TRAIL. Serial Fluc imaging revealed mNSC-SM7L/S-TRAIL induced a significant reduction in tumor volume that persisted through 4 weeks of treatment (Fig. 5F). Although mNSC-SM7L and mNSC-S-TRAIL also reduced GBM volumes, neither mono-therapy was as effective as the mNSC-SM7L/S-TRAIL combination treatment (Fig. 5F). These results were confirmed by post-mortem IHC that showed the significant reduction in tumor volumes by mNSC-SM7L/S-TRAIL (Fig. 5G), as well as an upregulation in cleaved caspase-3 as revealed by immunostaining (Fig. 5G).

## Discussion

Multifunctional molecules offer significant potential to improve treatment response and outcomes of patients suffering from cancer [1]–[3]. However, few DNA-based molecules exist that incorporate both highly functional therapeutic domains and diagnostic imaging domains. In this study we report the design and validation of SM7L, a unique multifunctional molecule with potent anti-tumor effects derived from enhancement of the therapeutic cytokine MDA-7/IL-24 and diagnostic properties due to incorporation of a photon-emitting luciferase. Used in combination with real-time non-invasive imaging and stem cell-based delivery, we showed the diagnostic domain of SM7L revealed important insights into the pharmacokinetics of SM7L therapy while the therapeutic domain markedly attenuated progression of highly malignant or highly invasive GBM as mono- or combination therapy. Together, these results demonstrate the efficacy and versatility of the multifunctional molecule SM7L for cancer therapy.

MDA-7/IL-24 is a distinctive cytokine that uniquely displays potent anti-cancer activity toward a broad-spectrum of human cancers, without provoking toxicity in normal cells or tissues [8], [9], [11], [12], [17], [28]. The molecule is composed of a central consensus sequence that has close homology to members of the IL-10 cytokine gene family, and a N-terminal secretion sequence that drives extracellular release [8], [9], [11], [12], [23], [24], [28]. The effects of extracellularly delivered MDA-7/IL-24 are mediated through interactions with IL-20Rα/β receptors on the cell surface, and the natural secretion of MDA-7/IL-24 leads to “bystander” effects via binding to surface receptors on adjacent and distant tumor cells resulting in stabilization, production and secretion of MDA-7/IL-24 by autocrine/paracrine processes [25], [26]. The primary goal of this study was to enhance the therapeutic potential of MDA-7/IL-24 by designing a new molecule that exploits the anti-tumor domain of wild-type MDA-7/IL-24. We theorized that this could be achieved by introducing diagnostic properties to serially monitor therapeutic levels and through enhanced secretion to increase extracellular levels and subsequent activation of IL-20Rα/β receptors. The resulting molecule, SM7L, is distinctive and contains the therapeutic domain of MDA-7/IL-24 flanked by the Flt-3 ligand signal sequence on the N-terminus and the diagnostic molecule Gaussia luciferase on the C-terminus. Predictive computer modeling suggested that the C-terminal fusion of MDA-7/IL-24 would allow MDA-7/IL-24 to retain efficient IL-20Rα/β binding, activity of the luciferase, and potentially increase molecular stability. Furthermore, replacement of the endogenous MDA-7/IL-24 secretion sequence with the signal sequence from the Flt-3 ligand was anticipated to achieve the desired enhancement in extracellular secretion. When the hypothetical molecule was engineered and tested, our results corroborated the predictive modeling. The optimized SM7L retained anti-tumor properties, robust luciferase activity, and the new signal sequence lead to enhanced secretion. The precise mechanisms by which the fusions in SM7L contribute to all of these increased activities are unclear, however previous data has suggested GST fusion to MDA-7/IL-24 increased the intracellular activity of the molecule, potentially via enhanced uptake and protein stabilization [29]. This finding suggests that the fusion of luciferase to MDA-7/IL-24 may function in a similar way to increase protein stabilization, but further research is required to prove this hypothesis. The enhanced anti-tumor toxicity of SM7L involves enhanced modulation of the ERK and MAPK signaling pathways (Fig. 2), key mediators of MDA-7/IL-24 tumor toxicity. In total, these results demonstrate the advantages of SM7L and the potential of utilizing predictive modeling to design optimized molecules that improve the anti-tumor efficacy of therapy treatments.

Our results show that a single administration of mNSC-SM7L significantly delayed peripheral and intracranial GBM progression. Previously, most MDA-7/IL-24 therapies were contingent on *ex vivo* transduction or multiple infusions of a replication incompetent Ad5. While this approach was shown to slow intracranial GBM progression [16], *ex vivo* infection of cells lacks clinical translatability and multiple Ad5 injections increase the complexity of the therapy and leads to activation of the host immune system thereby promoting neutralization of the therapeutic virus. Using the diagnostic properties of SM7L, a single mNSC-SM7L engraftment lead to highly localized and stable therapeutic delivery for days to weeks *in vivo*. In these experiments, we chose the method of delivery most commonly used in the clinics for stem cell or purified protein/chemotherapeutic delivery. The vast majority of pre-clinical studies have evaluated efficacy using direct intratumoral injection of therapeutic stem cells in GBM models [18], [30]–[32]. We have also shown that IV delivery of stem cells leads to significantly lower percentage of cells at the site of GBM compared to intratumoral injection [33], [34], and studies have shown the majority of stem cells accumulate in the lung following IV injection [33], [34]. Further demonstrating the clinical relevancy of intratumoral injection, the first clinical trial of stem cell-delivered targeted therapy for GBM was recently initiated at the City of Hope National Medical Center and uses local intratumoral stem cell injection. For these cumulative reasons, we chose to focus only on stem cells injected into the tumor and not IV. In contrast, many chemotherapies and purified protein therapies are administered systemically, either orally or IV. Therefore, we chose to investigate IV delivery of purified SM7L for comparison. Interestingly, our results showed a slight decrease in stem cell volumes and SM7L levels five days post-implantation. Although the precise underlying cause is unclear, we have previously observed a similar decrease of approximately 20% in stem cell volumes in the days following implantation [21]. We speculate that stem cell death may be due to small sub-populations of cells being damaged during implantation that then die shortly after injection, or some cells not being sustained by the brain microenvironment following implantation. Despite the cause of the cell death, we have never previously been able to detect the effect the decrease in stem cell volume has on therapeutic delivery. Our results show the decrease in SM7L parallels the decrease in stem cell volume, and the diagnostic properties of SM7L will allow optimization of dosing to maximize tumor cell killing in future studies. Accordingly, eliminating the need for multiple injections, coupled with the enhanced anti-tumor properties and diagnostic capacity of SM7L suggests that stem cell-delivered SM7L therapy will be superior to current MDA-7/IL-24 therapy for GBM when this cytokine is delivered using a replication incompetent Ad5.

Despite the promise of MDA-7/IL-24 and SM7L therapy, the results of this study and multiple other studies suggest that these mono-therapies primarily slow tumor cell growth and the remaining residual tumor has the potential for rapid tumor recurrence upon termination of the therapy. To improve therapeutic efficacy, MDA-7/IL-24 has been combined with cytotoxic and immune therapies for treatment of GBM [8], [9], [11], [12], [35], [36]. However, a limitation of the prior studies was the need for *ex vivo* manipulation to observe significant effects *in vivo*. Additionally, although the combinations improved therapeutic outcomes, the effects were insufficient and significant tumor burden was typically observed at the termination of therapy. In contrast, we observed the combination of mNSC-SM7L/TRAIL nearly eliminated tumor burden within 5 days of treatment, induced greater GBM killing than the combination of MDA-7/IL-24 and radiation, and was observed without pre-treatment of cells prior to implantation. The effectiveness of MDA-7/IL-24/TRAIL therapy has been reported in models of colorectal cancer [37], where co-administration of oncolytic adenovirus encoding MDA-7/IL-24 and TRAIL eradicated tumors in mice. In addition to dual activation of both the apoptotic cascade and the complex MDA-7/IL-24 signaling pathways, studies have suggested that MDA-7/IL-24 treatment further enhance the efficacy of concomitant TRAIL therapy by upregulating TRAIL death receptors or increasing the release of TRAIL from tumor cells [38]. This suggests the potential for synergistic cell killing by MDA-7/IL-24 and TRAIL therapy which was not observed in our study. While the full mechanism mediating MDA-7/IL-24 and TRAIL combination therapy remains to be elucidated, clearly the combination of MDA-7/IL-24 and TRAIL simultaneously target both cell proliferation and apoptosis pathways to increase GBM cell killing (Fig. 6) and may be a highly efficacious approach to treating GBM and potentially other malignant neoplasms.

To significantly improve management of aggressive neoplasms and enhance survival of patients with terminal cancers, new and more effective therapies are mandated. Building on the promising therapeutic cytokine MDA-7/IL-24, mNSC-SM7L provides specific advantages over replication incompetent Ad5 delivery of unmodified parental MDA-7/IL-24 that include superior anti-tumor efficacy, improved targeted delivery, and the ability to longitudinally track the therapeutic. When combined with simultaneous cytotoxic therapy, mNSC-SM7L/TRAIL potently eradicated malignant human GBM *in vivo*. In light of these advantages, we anticipate clinical application of stem cell-mediated delivery of SM7L holds promise to significantly benefit patients suffering from GBM and potentially other cancers.

## Materials and Methods

### Generation of Lentiviral Vectors Encoding MDA-7/IL-24 Fusion Variants

The lentiviral transfer plasmid CS-CGW [39], [40] was used as the backbone for all LV constructs. To create LV-MDA-7/IL-24 (M7), the cDNA sequence encoding MDA-7/IL-24 was PCR amplified using the vector porf-hil24 (Invivogen, San Diego CA) as a template. The restriction sites were incorporated into the primers, the resulting fragment was digested with *Nhe1* and *Xho1*, and ligated in-frame into *Nhe1/Xho1* digested CSCGW plasmid. To create LV-ssmda-7 (SM7), cDNA encoding the 60 bp signal sequence of Flt-3 ligand was amplified with primers including *Nhe1* and *BamH1* restriction sites using previously described DNA as template [41]. The cDNA encoding MDA-7/IL-24 without the endogenous signal sequence was amplified with primers containing *BamH1* and *Xho1* restriction sites. The two fragments were digested and ligated into CSCGW plasmid digested *Nhe1/Xho1*. To engineer the luciferase fusion variants, the cDNA encoding *Gaussia* luciferase (Gluc) was amplified using the pGluc-Basic vector (New England Biolabs, Ipswich MA) as a template with primers containing *EcoV* and *Xho1* restriction sites. Both LV-SM7 and LV-M7 vectors contained an *EcoV* site in the c-terminus of MDA-7/IL-24. Both vectors were digested with *EcoV* and *Xho1*, and the Gluc fragments were ligated in-frame. LV-Fluc-mCherry was kindly provided by Dr. Andrew Kung (Dana-Farber Cancer Institute, Boston MA). Lentiviral vectors were produced by transient transfection of 293 T cells as previously described [27].

### Cell Lines

Human glioblastoma cell lines U87-Fluc (a kind gift from Claire Sauvageot, Brigham and Women’s Hospital, Boston, MA) [42]. H4, and U251, as well as 293 T cells were purchased from American Type Culture Collection (ATCC, Manassas, VA) and cultured as previously described [1], [21], [43], [44]. Primary mouse neural stem cells were purchased from Stem Cell Technologies and cultured as previously described [1].

### Tumor Models and *In Vivo* Bioluminescent Imaging

In this study, several tumor models were utilized. In all studies, a 4 µl injection volume was used for tumor cells and a 6 µl injection volume was used for stem cells. All experimental protocols were approved by The Subcommittee on Research Animal Care at Massachusetts General Hospital, and care of the mice was in accordance with the standards set forth by the National Institutes of Health *Guide for the Care and Use of Laboratory Animals*, USDA regulations, and the American Veterinary Medical Association: 1) To determine the effects of stem cell-delivered SM7L on intracranial GBMs, 2×10^5^ U87-Fluc human glioma cells and 1×10^5^ mNSC expressing SM7L or control (GFP-Rluc) were implanted stereotactically into the right frontal lobe of SCID mice (from bregma: –2 mm lateral, −2 mm ventral). On days 1,3, 6, 8, 14, 22, 29, and 35 post-injection mice received an intraperitoneal injection of D-luciferin (2 mg; prepared in 150 µl of saline) and tumor progression was monitored by FLuc imaging. On day 29 post-implantation, a subset of mice from control (mNSC-GFP-Rluc) and mNSC-SM7L treatment groups were injected with coelenterazine (100 µg) and Rluc or Gluc imaging was performed to assess stem cell volumes or SM7L levels respectively. 2) To determine the effects of stem cell-based SM7L/S-TRAIL combination therapy on intracranial GBMs, 2×10^5^ U87-Fluc human glioma cells and 1×10^5^ mNSC expressing SM7L, S-TRAIL, SM7L/S-TRAIL or control (GFP-Rluc) were implanted stereotactically into the right frontal lobe of SCID mice (from bregma: –2 mm lateral, −2 mm ventral). Tumor progression was then monitored by FLuc imaging on days 1 and 29 as described above. All the other methods utilized in this study are described in detail in Methods S1 section.

### Statistical Analysis

Data are expressed as mean±SD and were analyzed by either Student’s T-test or ANOVA (after Bartlett’s test of homogeneity of variance), followed by the Newman-Keuls correction for multiple comparisons. Differences were considered significant at P<0.05.

## Supporting Information

Figure S1 **Levels of IL20α/β receptors in GBM cell lines.** (A) Relative expression of IL20α/β receptors on various GBM determined by RT-PCR. Experiments were performed in triplicate with mean and SD reported.
(TIF)

Figure S2 ***In vitro*** **characterization of stem cell-delivered SM7L on glioma line, U251.** (A) Representative images and summary graphs of cell viability assays performed on U251 established human GBM cells co-cultured with mNSC-GFP, mNSC-M7, or mNSC-SM7L. U251-Fluc-mcherry GBM cells were seeded in 96 well plates. 24 hrs later, control or mNSC secreting wild-type or SM7L were overlayed on the cells. Three and five days later, Fluc imaging was performed to determine the effects of stem cell-delivered M7 or SM7L on GBM cell growth. (B) Summary data demonstrating the viability of GBM8 primary patient-derived cells co-culutred with mNSC-GFP or mNSC-SM7L. Cell viability was determined by Fluc imaging 1, 3, and 5 days post-treatment. *p<0.05 vs. control. Experiments were performed in triplicate with mean and SD reported.
(TIF)

Methods S1 This section includes details on LV transduction, molecular modeling, caspase and viability assays, in vitro bioluminescence imaging and fluorescence microscopy, western blot analysis, quantitative RT-PCR, tumor models and *in vitro* and *in vivo* imaging.
(DOC)
