# Supplementary figures and images for: A First-Generation Multi-Functional Cytokine for Simultaneous Optical Tracking and Tumor Therapy
Source: PLoS One. 2012 Jul 11;7(7):e40234. doi: 10.1371/journal.pone.0040234 (PMC3394792; doi:10.1371/journal.pone.0040234)

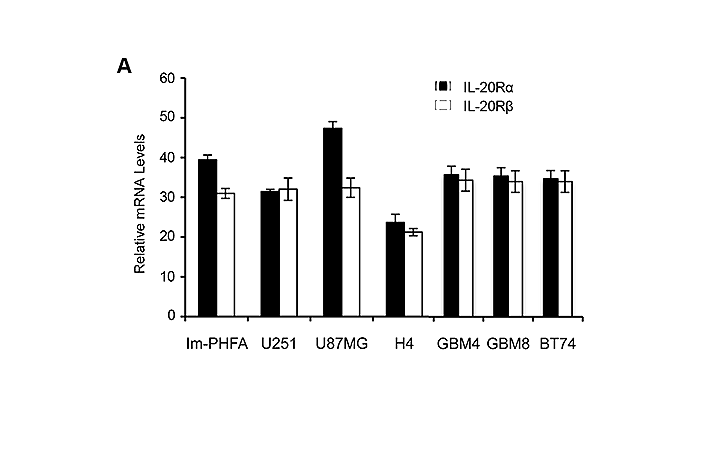

Supplement: Figure S1 — Levels of IL20α/β receptors in GBM cell lines. (A) Relative expression of IL20α/β receptors on various GBM determined by RT-PCR. Experiments were performed in triplicate with mean and SD reported. (TIF) [file pone.0040234.s001.tif]

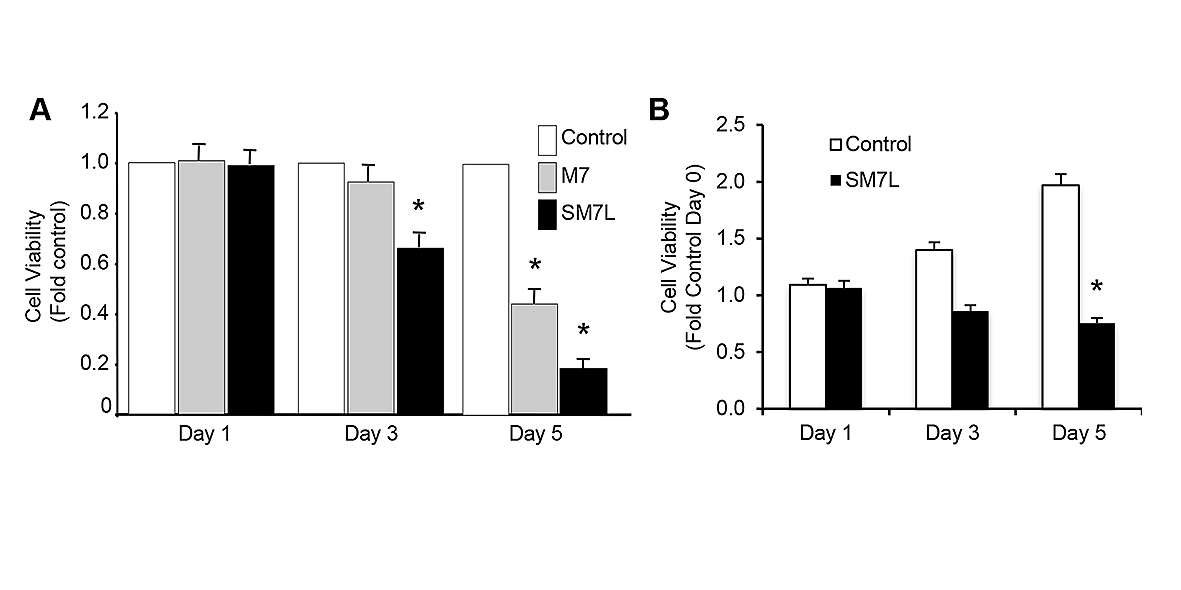

Supplement: Figure S2 — In vitro characterization of stem cell-delivered SM7L on glioma line, U251. (A) Representative images and summary graphs of cell viability assays performed on U251 established human GBM cells co-cultured with mNSC-GFP, mNSC-M7, or mNSC-SM7L. U251-Fluc-mcherry GBM cells were seeded in 96 well plates. 24 hrs later, control or mNSC secreting wild-type or SM7L were overlayed on the cells. Three and five days later, Fluc imaging was performed to determine the effects of stem cell-delivered M7 or SM7L on GBM cell growth. (B) Summary data demonstrating the viability of GBM8 primary patient-derived cells co-culutred with mNSC-GFP or mNSC-SM7L. Cell viability was determined by Fluc imaging 1, 3, and 5 days post-treatment. *p<0.05 vs. control. Experiments were performed in triplicate with mean and SD reported. (TIF) [file pone.0040234.s002.tif]
