# Supplementary material for: A First-Generation Multi-Functional Cytokine for Simultaneous Optical Tracking and Tumor Therapy
Source: PLoS One. 2012 Jul 11;7(7):e40234. doi: 10.1371/journal.pone.0040234 (PMC3394792; doi:10.1371/journal.pone.0040234)
Supplement: Methods S1 — This section includes details on LV transduction, molecular modeling, caspase and viability assays, in vitro bioluminescence imaging and fluorescence microscopy, western blot analysis, quantitative RT-PCR, tumor models and in vitro and in vivo imaging. (DOC) [file pone.0040234.s003.doc]

**SUPPLEMENTAL MATERIALS AND METHODS**

**Lentiviral transduction:** 293T, H4, U87, or mNSC were transduced with control virus or mda-7 variants at an MOI of 2 (glioma lines, 293T) or 5 (mNSC) in a growth medium containing 4 μg/mL protamine sulfate and visualized for green fluorescent protein (GFP) expression by fluorescence microscopy 24-48 h post-transduction. U87, U251, and GBM8 were transduced with LV-mCherry-Fluc as described above using an MOI 2.

**Molecular Modeling:** Homology modeling was used to construct a model of the wild type-mda-7 dimer bound to the IL-20Rαβ receptor dimer. The complex of the IL-10 dimer bound to the IL-10R1 receptor (PDB code 1y6k), downloaded from the Protein Data Bank , was used as the primary template. The level of global (Smith-Waterman) similarity between the modeled portion of MDA-7/IL-24 (residues 53-206) sequence and the IL-10 template sequence is 62%, with 22% amino acid identity. The modeling was carried out using Version 9v7 of the MODELLER program with symmetry restraints placed on the main chain atoms of the subunits. Both the IL-20Rα and IL-20Rβ receptor models were based on the IL-10R1 structure, also from the IL-10/IL-10R1 complex (PDB code 1y6k). We modeled only the extracellular domains of the receptors, residues 51-240 and 32-227 for IL-20Rα and IL-20Rβ respectively. The measures of sequence similarity and identity are 62.6% and 25.1% for IL-20Rα and 53.1% and 27.0% for IL-20Rβ. Since the IL-10/IL-10R1 structure includes only the α chain of the receptor, the relative orientation of the two chains in the IL-20Rαβ receptor model was based on the IL-6/IL-6R complex (PDB code 1p9m). IL-6R has slightly lower sequence similarity and identity to IL-20Rα (41.6% similarity and 26.8% identity) and to IL-20Rβ (39.2% similarity and 26.1% identity) than IL-10R1 but it yields a clash-free model of the IL-20Rα/β receptor bound to the IL-10 dimer. Two copies of the 20Rα/β receptor were then placed relative to the mda-7 dimer model using the position of the IL-10R1 receptor relative to IL-10 in the IL-10/IL-10R1 complex as the template. Clashes were resolved using the minimization protocol in the ClusPro server .

The wild type-mda-7 structure was optimized by adding a c-terminal luciferase fusion domain. The *Gaussia* luciferase structure was modeled using the I-TASSER server . The top I-TASSER model was docked to itself using the dimer docking mode of the ClusPro server . The models generated by ClusPro were filtered based on knowledge of the positions of the termini in the model of the mda-7 dimer. The mda-7 dimer and the model of the luciferase dimer were all linked using MODELLER with main chain symmetry restraints. Thus, the resulting construct consists of the mda-7 dimer covalently linked to a dimer of the luciferase fusion domain. The luciferase is expected to contribute to the improved stability of the dimer. It was shown that when bound to the IL-20Rα/β receptor dimer with the mda-7 dimer, the modified mda-7 construct does not clash with the receptor.

**Caspase and viability assays:**

*Investigating SM7L:* To determine the effects of SM7L, U87 or H4 GBM cells were seeded in 96-well plates (1x10^4^ cells/well). Twenty-four h after plating, the cells were transduced with LV encoding SM7L, M7, or control. 5 days post-transduction, cell viability was determined by MTT assay as described previously . To determine the effects of stem cell delivered SM7L on glioma viability, U87, U251, GBM8 cells (2x10^3^) expressing Fluc-mCherry were seeded in 96 well plates. 24 h later, mNSC (2x10^3^ cells) transduced with LV-M7, LV-SM7L, or control were overlayed on the tumor cells. On days 1, 3 and 5 post-treatment, cells were incubated in 100 μg/ml D-luciferin and glioma cell viability was determined by assaying luciferase activity in a luminometer (Bio-rad, Hercules, CA).

*Investigating SM7L and S-TRAIL combination therapy:* To investigate the effects of radiation, SM7L, or S-TRAIL alone or in combination, U87 and U251 glioma cells (2x10^3^) were seeded in 96 well plates. Cells were then irradiated (4 Gy), treated with SM7L-conditioned media, or left untreated. 48 h post-treatment, a subset of cells from each group were treated with conditioned media containing S-TRAIL. Twenty-four h later, cell viability was determined by luciferase-based assay. To determine the effects of combined stem cell-delivered SM7L/S-TRAIL on glioma cell viability, U87-FLuc and U251-Fluc glioma cells were co-cultured with control mNSC, mNSC-M7 or mNSC-SM7L as described above. On day 4 of co-culture, mNSC-S-TRAIL (2x10^3^ cells) were seeded into a subset of SM7L-treated wells. Glioma cell viability was determined 24 h post-mNSC-S-TRAIL treatment by luciferase assay. To assay caspase 3/7 activity following SM7L and S-TRAIL treatment, U87 and U251 glioma cells were treated with control or SM7L as described above. After 4 days of treatment, a subset of each treatment type was additionally incubated with S-TRAIL (200 ng). Caspase 3/7 activity was determined using CaspaseGLo 3/7 assay (Promega) 18 h later.

***In vitro* bioluminescent imaging and fluorescence microscopy:** To investigate the extracellular levels of SM7L, 0-1x10^5^ 293T were seated in 96-well plates and transduced with equal MOI of LV encoding M7L, SM7L, or M7. 24 h post-transduction, media was changed and replaced with 50 μls of culture medium. 24 h later, media was collected, transferred to separate 96-well plate, and cells or media were incubated with 15 μg of coelenterazine for 10 min, and photon emission was measured using a cryogenically cooled high efficiency CCD camera system (Roper Scientiﬁc, Trenton, NJ). To confirm a linear correlation between cell number and Gluc photon emission, increasing numbers of mNSC (0.1-2x10^4^ cells) expressing LV-SM7L were seated for 24 h in 96 well plates, at which time cells were incubated with 15 μg of coelenterazine for 10 min, and photon emission was measured using a luminometer. To determine the duration of transgene expression, mNSC were transduced with SM7L. On days 1, 3, 5 and 10 post-transduction, cells (1x10^5^ cells) were collected, combined with coelenterazine (15 μg), luciferase activity determined using a luminometer, and expressed relative to cell number determined by cell viability assay.

**Western blot Analysis:**

To determine the effects of M7 or SM7L on GBM signaling, cell lysates were collected from U87 GBM cells treated with control, M7 or SM7L containing media following 5 days of treatment. Lysates were resolved by SDS-PAGE, transferred to nitrocellulose, and probed with antibodies against phosphorylated p38MAPK, total p38MAPK, phosphorylated ERK, or total ERK (Cell Signaling Technologies, Danvers MA). To investigate the molecular mechanisms mediating SM7L and S-TRAIL co-treatment, U87 and U251 cells (2x10^5^) were treated with control or SM7L containing media. On day 4 post-treatment, cells were either left untreated or incubated with S-TRAIL containing media. 18 h post-S-TRAIL treatment, lysates were collected and subjected to Western blot analysis with antibodies against phosphorylated p38MAPK, total p38MAPK, phosphorylated ERK, total ERK, cleaved PARP, total caspase-8, and cleaved caspase-8 (Cell Signaling Technologies). All blots were quantified using NIH Image (National Institute of Health, Bethesda, MD).

**Quantitative Real-Time PCR:** Determination of IL-20Rα and IL-20Rβ was performed as described previously . Briefly, total RNA was isolated and reverse transcribed from various glioma cell lines. Quantitation of IL-20Rα and IL-20Rβ was performed on cDNA prepared from total RNA using primers specific for IL-20Rα and IL-20Rβ. Standard ampliﬁcation conditions were utilized according to the manufacturer’s speciﬁcations.

**Tumor Models and *In Vivo* Imaging:** In this study, several tumor models were utilized. All experimental protocols were approved by The Subcommittee on Research Animal Care at Massachusetts General Hospital, and care of the mice was in accordance with the standards set forth by the National Institutes of Health *Guide for the Care and Use of Laboratory Animals*, USDA regulations, and the American Veterinary Medical Association. In all studies, FLuc imaging was performed by injecting mice with 2 mg of luciferin via i.p., and imaging was performed 10 minutes after substrate injection using 7 minutes of acquisition time. When necessary, mice were shaved at the time of initial surgery and once each week until termination of the experiment. Acquisition of bioluminescent images was followed by capture of a light image in the chamber using dim polychromatic illumination. Following data acquisition, post-processing and visualization was performed using a in house program, CMIR Image, using image display and analysis suite developed in IDL (Research Systems Inc., Boulder, CO, USA). Regions of interest were defined using an automatic intensity contour procedure to identify bioluminescence signals with intensities significantly greater than the background. The mean, standard deviation, and sum of the photon counts in these regions were then calculated. For visualization purposes, bioluminescence images were fused with the corresponding white light surface images in a transparent pseudocolor overlay, permitting correlation of areas of bioluminescent activity with anatomy.

1) To investigate the pharmacokinetics of SM7L delivered by mNSC, 4x10^6^ U87-Fluc glioma cells cells were implanted subcutaneously in mice. Twenty-four h later, mice were injected with 2 mg D-luciferin and FLuc imaging was performed to identify tumor location. Twenty-four h after FLuc imaging, mNSC expressing SM7L were implanted around established tumors. GLuc imaging was performed as described to monitor SM7L levels at 0 and 24 h following injection of 100 μg of coelenterazine prior to each imaging session. In a subset of mice, animals were sacrificed 1 h after injection of coelenterazine, and ex vivo imaging was performed on extracted lungs, liver, kidney, blood, tumor, and heart. The tissue was weighed and data was expressed relative to tissue weight. In a separate set of mice, FLuc imaging was performed 0, 2, 7, and 10 days post-mNSC injection to determine the effects of stem cell-delivered SM7L on growth of established tumors. To investigate the kinetics of intravenous SM7L delivery, mice were implanted with 4x10^6^ U87-Fluc glioma cells. Twenty-four h later, mice were injected with 2 mg D-luciferin and FLuc imaging was performed to identify tumor location. Twenty-four h later, mice were injected with media containing SM7L by intravenous infusion. Five minutes post-injection, mice were injected with 100 μg of coelenterazine and imaged every 5 minutes for 50 minutes, and again at 24 h post-SM7L infusion following re-injection of coelenterazine. In a separate set of mice, animals were sacrificed 1 h post-media injection, the liver, lung, tumor kidney, urine, and blood were collected and imaged ex vivo. The tissue was weighed and data expressed relative to tissue weight. In a separate set of mice, FLuc imaging was performed 1, 3, 7, and 10 days post-media injection to determine the effects of intravenous SM7L on subcutaneous tumor growth. In both NSC-SM7L and IV-SM7L, background regions were chosen by selecting 5 non-tumor bearing regions of the mouse determined by comparison with Fluc tumor images. Data was then expressed by dividing the SM7L signal at the tumor regions by the signal from these non-tumor background regions.

2) To determine which events in stem cell-mediated delivery of SM7L could be tracked by in vivo imaging, U87Fluc-mCherry (3x10^6^ cells) were implanted in the upper tissue layer of dorsal skin fold window chambers as described previously . Three days later, mNSC-SM7L or mNSC-GFP (2x10^6^ cells) were implanted around the established tumors. Mice were then imaged on day 1 and day 5 by dual bioluminescence imaging or subjected to intravital microscopy as described .

**Tissue processing:** Immediately following the last imaging session in mice implanted with control or SM7L-secreting stem cells, mice were perfused, brains were removed, and 30 μM sections of the brains were generated using a vibratome. Floating brain sections were immunostained with antibodies against human Ki67 (DAKO, Carpinteria, CA), followed by incubation with Alexa dye 555 nm secondary antibodies. GFP-expressing stem cells and Ki67 immunostaining was visualized by confocal microscopy as described previously. To visualize the effects of mNSC-GFP-Rluc or mNSC-SM7L/S-TRAIL, mice were sacrificed on day 3, and processed as described above. Floating sections were stained with H&E, or probed with antibodies caspase-3 followed by incubation with Alexa dye 555 nm secondary antibodies and confocal microscopy. Caspase-3 activation was assessed by manually counting the number of caspase-3-positive cells in each treatment group.

**REFERENCES**

1. Berman HM, Westbrook J, Feng Z, Gilliland G, Bhat TN, et al. (2000) The Protein Data Bank. Nucleic Acids Res 28: 235-242.

2. Smith TF, Waterman MS (1981) Identification of common molecular subsequences. J Mol Biol 147: 195-197.

3. Fiser A, Sali A (2003) Modeller: generation and refinement of homology-based protein structure models. Methods Enzymol 374: 461-491.

4. Comeau SR, Gatchell DW, Vajda S, Camacho CJ (2004) ClusPro: an automated docking and discrimination method for the prediction of protein complexes. Bioinformatics 20: 45-50.

5. Roy A, Kucukural A, Zhang Y (2010) I-TASSER: a unified platform for automated protein structure and function prediction. Nat Protoc 5: 725-738.

6. Yacoub A, Mitchell C, Lister A, Lebedeva IV, Sarkar D, et al. (2003) Melanoma differentiation-associated 7 (interleukin 24) inhibits growth and enhances radiosensitivity of glioma cells in vitro and in vivo. Clin Cancer Res 9: 3272-3281.

7. Hingtgen SD, Tian X, Yang J, Dunlay SM, Peek AS, et al. (2006) Nox2-containing NADPH oxidase and Akt activation play a key role in angiotensin II-induced cardiomyocyte hypertrophy. Physiol Genomics 26: 180-191.

8. Tannous BA, Kim DE, Fernandez JL, Weissleder R, Breakefield XO (2005) Codon-optimized Gaussia luciferase cDNA for mammalian gene expression in culture and in vivo. Mol Ther 11: 435-443.

9. Hingtgen SD, Kasmieh R, van de Water J, Weissleder R, Shah K (2010) A novel molecule integrating therapeutic and diagnostic activities reveals multiple aspects of stem cell-based therapy. Stem Cells 28: 832-841.

10. van Eekelen M, Sasportas LS, Kasmieh R, Yip S, Figueiredo JL, et al. (2010) Human stem cells expressing novel TSP-1 variant have anti-angiogenic effect on brain tumors. Oncogene 29: 3185-3195.

11. Shah K, Hingtgen S, Kasmieh R, Figueiredo JL, Garcia-Garcia E, et al. (2008) Bimodal viral vectors and in vivo imaging reveal the fate of human neural stem cells in experimental glioma model. J Neurosci 28: 4406-4413.
